# The Black Pill: New Technology and the Male Supremacy of Involuntarily Celibate Men

**DOI:** 10.1177/1097184X211017954

**Published:** 2021-05-18

**Authors:** Kayla Preston, Michael Halpin, Finlay Maguire

**Affiliations:** 1 Department of Sociology, 7938 University of Toronto, Toronto, Ontario, Canada; 2 Department of Sociology and Social Anthropology, 3688 Dalhousie University, Halifax, Nova Scotia, Canada; 3 Faculty of Computer Science, 3688 Dalhousie University, Halifax, Nova Scotia, Canada

**Keywords:** hegemonic masculinity, incels, technology, social media, online, communities, misogyny

## Abstract

Involuntary celibates, or “incels,” are people who identify themselves by their inability to establish sexual partnerships. In this article, we use analytic abduction to qualitatively analyze 9,062 comments on a popular incel forum for heterosexual men that is characterized by extensive misogyny. Incels argue that emerging technologies reveal and compound the gender practices that produce involuntarily celibate men. First, incels argue that women’s use of dating apps accelerates hypergamy. Second, incels suggest that highly desirable men use dating apps to partner with multiple women. Third, incels assert that subordinate men inflate women’s egos and their “sexual marketplace value” through social media platforms. We argue that incels’ focus on technology reinforces essentialist views on gender, buttresses male domination, dehumanizes women, and minimizes incels’ own misogyny. We discuss findings in relation to theories of masculinity and social scientific research on the impacts of emerging technology.

## Introduction

Involuntarily celibates, or “incels,” are people who define themselves by their inability to have sexual intercourse and romantic relationships. Although the term “incel” was coined by a woman (Taylor 2018), incels are predominately men seeking heterosexual sex. Their communities and interactions are online. Incel discourse is characterized by anti-feminism, endorsing violence against women, removal of women’s rights, self-loathing, and racism (Chang 2020; Jaki et al. 2019). Incels have committed several mass homicides, including Elliot Rodger’s killing of six people in Isla Vista, California (Witt 2020), and Alek Minassian’s murder of 10 people in Toronto, Canada (Brockbank 2019). In this article, we provide the first analysis of a popular incel forum, describing how incels identify emerging technologies (i.e., dating apps and social media) as revealing and amplifying the gender practices that produce men who are involuntarily celibate.

Incels situate their experiences as emblematic of the growing social problems facing men. There is some evidence for the problems that incels highlight. For instance, men are experiencing decreased participation in post-secondary education and increasing social isolation (DiPrete and Buchmann 2013; Vespa, Lewis, and Kreider 2013). Similarly, there is a downward trend in men’s reported sexual activity, with an increasing number of men reporting that they have not had any sexual activity (see Ingraham 2019 for a summary). While these trends are important, men nonetheless continue to experience considerable social advantages (Besen-Cassino 2019; Martin 2001). In this sense, incels’ arguments orient to a perceived decrease in the patriarchal dividend, which refers to men’s disproportionate benefit from unequal gender relations (Connell 2000).

Aside from incels, many online communities offer explanations for the challenges and problems facing men, invoking lay interpretations of evolutionary psychology, economics, and genetic science (Ging 2019; Nagle 2017; Van Valkenburgh 2018). This article contributes to this literature by demonstrating how incels buttress the masculine order with a form of gendered technological determinism, which lays blame for their inability to establish sexual relationships on the impact that emerging technologies have on gender relations. We detail incels’ arguments that dating apps (e.g., Tinder^™^) facilitate hypergamy, that dating apps favor dominant men (called “Chads”), and that men incels perceive as subordinate (called “normies,” “betamales,” or “numales”) use social media to inflate women’s egos. We argue that incels’ focus on technology excuses their misogyny, as they situate themselves as the hapless victims of a new, technology-mediated dating market. Their assessment maintains male dominance and denigrates women, as incels argue it is the inequitable allocation of sexual access to women amongst men, rather than dehumanizing and objectifying gender relations, which needs to change.

## Background

### Incels and the “Manosphere”

The term “incel” was coined in 1997 by Alana (last name withheld), a Canadian lesbian woman who maintained a blog on her experiences with celibacy and sexuality (Taylor 2018). Alana’s blog received awards from the LGBT community and was part of a growing number of websites addressing celibacy. In 2003, Alana became distressed by the increasing negative nature of the blog’s online discussions and shut down her website (Taylor 2018). In addition to Alana’s site, incels argue that 1990s websites on shyness and seduction were formative for the community, while more recent discussion boards on Reddit and 4Chan tied the community to misogyny (Incels.co 2020a). Indeed, Chang’s (2020) analysis of 14 threads on a now-defunct Reddit board for incels details how their ubiquitous use of the term “femoid” (female humanoid or female android) constructs women as objects and facilitates violence and hostility toward women. Accordingly, a community started by a woman for a general experience transitioned into one that is dominated by misogynistic heterosexual men.

As incel communities changed, so did the criteria for being an incel. For instance, Alana’s website defined incels broadly, including people who never dated, those with little dating experience, and those who struggle to establish relationships due to some issue they cannot control (e.g., shyness) (Incels.co 2020a). Likewise, not all celibate men identify as incels, as Terry (2012) demonstrates in an interview-based study of nine men that still desired sex but exercised self-control by practicing celibacy. The site we analyze defines “incel” as anyone who is, “rejected by the members of the sex they are sexually attracted to” (Incels.co 2020b). Despite the openness of their definition of “incel,” the community explicitly excludes women, individuals who deny incel tenets, and members who lose their incel status (Incels.co 2020b). Consequently, incel identity is increasingly oriented to the experiences and perspectives of heterosexual men. Indeed, a deep learning analysis of the now-defunct incels.me site suggests users are heterosexual, male, under the age of 20, and see themselves as ugly (Jaki et al. 2019).

Incels are part of a group of male-oriented websites that are collectively referred to as the “manosphere.” Some manosphere groups have practices that are contrary to those of incel communities, such as pick-up artist sites where men learn seduction tips, and “men go their own way” (MGTOW) communities, wherein men abandon the pursuit of marriage due to overly punitive divorce laws. For example, Van Valkenburgh’s (2018) qualitative analysis of 26 documents central to The Red Pill subreddit demonstrates how men on the forum view women as economic commodities and sexual success results by using strategies from economics and evolutionary psychology. Likewise, Ging’s (2019) 6-month analysis of multiple manosphere sites focusing on gamer culture, MGTOW, men’s rights groups, and pickup artists, suggests manosphere discourse aligns with hybrid masculinity (Bridges and Pascoe 2014), wherein dominant men selectively adopt practices of subordinate groups. Here, Ging (2019) argues that manosphere discourse mixes claims of apparent tolerance for homosexuality with a continued emphasis on gender inequality and male domination.

Despite some differences, manosphere sites are united in their anti-feminist agenda (Nagle 2017) and the belief that feminism has undermined men’s social role, resulting in systemic misandry (prejudice toward men). Indeed, Marwick and Caplan (2016) trace the use of “misandry” across the manosphere since the 1990s, observing that the term is used to build community while discrediting and vilifying feminism. Many manosphere communities also consider themselves to be “red pilled,” which references a scene from The Matrix wherein the protagonist is offered a red pill that will reveal the truth about the world. In the manosphere, accepting the red pill means awakening to the perceived destructive force of feminism (Van Valkenburgh 2018). In addition to red pill language, incels discuss the “black pill,” which means accepting the fact that “it’s over” and “inferior” men have no chance of ever establishing sexual relationships with women. As such, the community is defined by the relationship between heterosexual sex and masculine status.

### Society and Emerging Technology

The social impacts of dating apps are a central concern for incels. Research suggests that dating apps have altered dating practices, creating new spaces for sexual and romantic relationships, and considerably expanding an individual’s number of potential partners (Hobbs, Owen, and Gerber 2017). Social scientific and app developers’ in-house research indicates that these apps do introduce or amplify several biases (Gavin, Rees-Evans, and Brosnan 2019). For example, apps have demonstrable racism and racial bias (Mason 2016; Rudder 2009) and privilege physically attractive members (Hobbs, Owen, and Gerber 2017). Apps can also compound misogynistic behavior, as men exhibit aggressive strategies to obtain dates from women and respond with hostility when these strategies are unsuccessful (Shaw 2016). A systematic review (Abramova et al. 2016) further notes that women receive more contacts than men, while men start more conversations and receive fewer replies on dating apps. As we detail below, incels emphasize that apps are problematic because they diminish men’s power in sexual/romantic interactions.

Social media platforms are changing how individuals interact and perceive themselves. For instance, research suggests Instagram^™^, a photo-sharing platform, alters how individuals view and criticize their bodies in relation to the attention they do or do not receive (Butkowski, Dixon, and Weeks 2019), while both Facebook^™^ and Instagram^™^ exacerbate individuals’ pursuit of peer validation and facilitate the release of personal information to impress and attract followers (Yau and Reich 2019). Social media is often tied to bullying, with one study observing 9.76 million cases of bullying on Twitter over a two-year period, which includes both cyberbullying and sharing videos of offline bullying (Bellmore et al. 2015).

Popular discussion boards (e.g., Reddit, 4chan) are sites of online misogyny and antipathy toward feminism (Massanari 2017; Taylor and Jackson 2018; Van Valkenburgh 2018). The claims that men make about women on these sites have online and offline impacts on women. Jane (2018, 2014) demonstrates that women encounter considerable gender-based “cyberhate” and often have little recourse for reducing social media harassment, while Moloney and Love (2019) detail how men engage in gendertrolling and other forms of online oppression. Incels’ participation in and celebration of these misogynistic activities is well-documented, as they harass, degrade, objectify, and encourage violence against women (Chang 2020; Jaki et al. 2019). As we demonstrate below, incels connect these platforms to inceldom.

### Masculinity and Online Communities

Connell’s (1995, 2000 with Messerschmidt 2005) masculinity theory is central to many analyses of the manosphere and is likewise informative for our analysis of the incel community. Connell (1995) outlines a masculine order that centers around a dominant form of masculinity, referred to as hegemonic masculinity. Other forms of masculine practice are positioned in relation to hegemonic masculinity, with Connell (1995) identifying men who are not hegemonic but benefit from male dominance (complicit masculinity), men who are subordinated by dominant groups (subordinated masculinity), and men who occupy socially marginalized groups (marginalized masculinity). Connell’s (1995) theory emphasizes change and fluidity in hegemonic actions but maintains that hegemonic masculinity is characterized by the subordination of women (see also Connell and Messerschmidt 2005; Messerschmidt 2019).

Using Connell’s theory to analyze Reddit’s The Red Pill forum, Van Valkenburgh (2018) suggests that men display hegemonic behaviors while exchanging information about how to successfully seduce women. Contrastively, in a discussion of 4Chan.org, Nagle (2016) argues that applying hegemonic masculinity theory to incels and other “betamales” is inaccurate and tautological, as a “great deal” of their activities runs counter to hegemonic descriptions (e.g., enjoying My Little Pony). Ging (2019) introduces additional theoretical complexity, suggesting manosphere discourse exemplifies hybrid masculinity—men’s selective adoption of subordinate practices (Bridges and Pascoe 2014; Messner 2007) as they appear to adapt to emerging gender relations yet still explicitly focus on defeating feminism. Although incels might not consider themselves “alphas” or beneficiaries of male domination (e.g., Nagle 2016), we argue that their apparent “beta” masculinity orients to hegemony, as they suggest new technologies create sexual marketplace distortions that result in the unfair allocation of women among men. Here, incels argue that new technologies reveal and compound gender practices, which drive women toward a select group of attractive men. Incels do not participate in intimate relations with women but their explanations for this issue allow them to hold fast to notions of male supremacy and participate in the denigration, humiliation, and subordination of women. Their focus on technology and dating life also allows them to situate themselves as the victims of emerging social dynamics while justifying their misogyny.

## Method

Social scientists increasingly advocate systematic study of online communities (Baele, Brace, and Coan 2019; Nagle 2017; O’Neill 2018; Jane 2018; Ging 2019; Schmitz and Kazyak 2016; Van Valkenburgh 2018). We align with these researchers, arguing that online analyses are ideal for investigating the incel community. There are challenges for such analyses, as incels’ comments might be performative, insincere, or shaped by social desirability bias. Likewise, incels might highlight their perceived subordination while bracketing their privilege, as we observed multiple incels referencing advantaged social backgrounds (e.g., degrees at prestigious schools). Nonetheless, discussion board analysis is an ideal approach for investigating this community, particularly as members stated they did not discuss their inceldom in everyday life.

We analyzed the most popular English-language incel discussion board (Incels.co), wherein users post questions, comments, or news items, to discuss with other members. As of our analysis, the site had approximately 11,800 members, who generated 214,856 threads and spent a total of 665,112 hours on the website (see Incels.co for current statistics). In 2019 (Incels.co 2020c), the site conducted an in-house demographic survey (N = 400). Most users (59%) reported being “middle class,” while 34% were “lower class” and 7% “upper class.” Most users (50%) had completed high school, while 39% had completed college. About 50% of users are in the United States, while 56% described themselves as “White.” The remaining users reported a range of racial-ethnic backgrounds.

For this study, we qualitatively analyzed 9,062 comments sampled during 3 months in 2019. We selected the most popular threads to focus our analysis on content that generated high user engagement and to eliminate threads with little or no responses. The first and second authors read and re-read threads, iteratively developing operational definitions for codes and developing a coding tree. For instance, we detailed incels discussion of “sexual marketplace value” by using the code “women’s sexual marketplace decisions” to code women’s apparent preference for “Chads,” while women’s perceived control of dating is coded as “women’s sexual marketplace power.” Rather than use inter-coder ratings, we resolved coding differences through bi-weekly discussions and by refining our operational definitions of codes. We also exchanged and re-coded 15% of our threads to ensure we consistently applied codes. We produced 66 codes including: “incel-as-a-social-problem,” “dating apps,” “women-as-objects,” “women’s sexual market power,” and “women’s sexual market decisions,” which were used in this analysis. We used the NVivo™ qualitative software to assist coding.

We analyzed data using analytic abduction (Tavory and Timmermans 2014). Abduction is a method of inquiry (like induction and deduction) that focuses on surprising findings. Similar to induction, abduction reasons from empirical data rather than deductively investigating hypotheses. In contrast to inductive approaches, abduction de-emphasizes generating new concepts and instead emphasizes how novel findings might be used to interrogate, explore, or refine established theories. Accordingly, we focus on incels’ arguments that new technologies are fueling the inceldom and, despite their self-perceived subordinate status, how their arguments bolstered masculine hierarchies and male domination. We discuss findings in relation to Connell’s (1995) masculinity theory and hybrid masculinity (Bridges and Pascoe 2014).

## Findings

Incels argue that social media (e.g., Instagram^™^) and dating apps (e.g., Tinder^™^) reveal and compound the gender practices that create incels. We identify three key themes in their arguments (*Hypergamy and Dating Apps, Chads and Tinder, Betamales*, *and Instagram*). Here, incels depict women as commodities and discuss how new technologies produce sexual marketplace distortions, ultimately they assert that technology leads to an “unfair” allocation of sexual access to women among men. By focusing on technology, incels support male domination and legitimate their misogynistic discourse.

### Hypergamy and Dating Apps

Incels argue that dating apps are a driving factor in producing men who are involuntarily celibate. According to this logic, apps like Tinder^™^ and Bumble^™^ provide women with a large number of men for romantic/sexual partnerships. Incels see women as inherently hypergamous, which means they use these apps to constantly “trade up” and obtain a more desirable mate. Incels claim that dating apps compound hypergamy, unfairly excluding average and below-average men from romantic and sexual partnerships.

Incels suggest that dating apps provide women with a large pool of potential mates (or “matches”). On these apps, users view one another’s profiles, “swiping right” on users they are interested in and “swiping left” on users they are not interested in. Outside of incel forums, many dating app users have shared their match statistics, which anecdotally show that women are more discerning than men. For instance, one male user reports making approximately 27,000 right swipes over a 4-year period, yielding 110 matches, matching with 0.4% of the women he was interested in (Reddit.com 2019a). In contrast, a female user reports that she made approximately 3,700 right swipes over 13 months, resulting in nearly 2,500 matches, matching with 68% of the men she was interested in (Reddit.com 2019b). Incels use these statistics to advance arguments about hypergamy, suggesting that men are lucky to obtain any matches, while any woman can select the best partner from many men.

Incels argue that dating apps facilitate hypergamy because they create an environment wherein women do not select their “looksmatch,” which is an incel term for partnering with someone of comparable attractiveness. Incels claim that women use dating apps to “date up” by selecting men who are more physically attractive than they are. Thus, they argue women of average attractiveness can select men who are extremely attractive, rather than dating a man who is also of average attractiveness. Such mismatched selections can be identified because incels ubiquitously quantify physical attractiveness (see also Van Valkenburgh 2018). Incels rate people from 1 to 10, where 1 is very unattractive and 10 is very attractive. Incels suggest that dating apps make it possible for a woman that is “five” to partner with men who are “nines and tens.” As one user states:

I was often attracted to ugly girls around my looksmatch but every one of them rejected me because they could find better using tinder and shit like that…. I was called “ugly” even by girls WAY MORE ugly than me. Like 2/10 obese roasties.

In addition to calling these women ugly and obese, he uses the term “roastie,” referring to a misogynistic and shaming incel belief that promiscuous women develop elongated labia that resemble slices of roast beef. This user is arguing he was unable to establish a relationship with women who were overweight, physically unattractive, and promiscuous because such women have access to more desirable men than him through dating apps. As such, incels argue that apps create a dating context wherein all women have access to very attractive men and less attractive men cannot partner with their “looksmatch,” which unfairly relegates them to inceldom.

While incels focus primarily on hypergamy relative to physical attractiveness, they also argue that dating apps compound status-based hypergamy. Incels claim that women who they deem to be average or below average attractiveness are able to partner with men who are both attractive and socially successful:

Nowadays, foids want good looks [and] good income [and a good] personality. And they are getting it, make no mistake about it. Because of Tinder and hypergamy. Foids prefer to share a Chad rather [then] settle with anything less.

Here, “foid” is shorthand for “femoid,” an incel term comparing women to androids, which incels themselves state is a “derogatory term” to suggest that “females are not fully human” (Incel.co. 2020d; see also Chang 2020). This user complains that “foids” have high standards for their sexual partners, as they want men who are attractive, wealthy, and have pleasant personalities. While these might seem like mundane criteria, many incels see themselves as failing in all three categories. As incels perceive these requirements as difficult or impossible to obtain, they are effectively “ruled out” of romantic life due to women’s hypergamy and modern dating technology. Indeed, as this user suggests, women would rather “share a Chad” (i.e., be in a polyamorous relationship) than date an incel.

In this section, we demonstrated how incels perceive dating apps as revealing and compounding women’s innate hypergamy. Incels situate themselves as hapless victims in this scenario, seeing themselves as average or below average men who are excluded from romantic life due to a confluence of external forces, which leaves the role of their prolific and caustic misogyny unexamined. Here, incels victimhood is firmly tied to their ideas that they are owed sex and entitled to women’s bodies. While incels connect heterosexual sex to masculine status (Connell 1995; Pascoe 2011; Taylor and Jackson 2018), they argue that it is apps and women that deny them this status. Incels denigrate women for rejecting them while depicting women as simplistically and hedonistically pursuing the most physically desirable men. While apps are a driving factor in the inceldom, it is women that receive hate and anger.

### Chads and Tinder

In tandem with increasing hypergamy, incels argue that dating apps provide women with access to “Chads,” an incel-term used to describe the most attractive men. In general, they define Chads as white, tall, and muscular, with attractive faces and well-defined jawlines, with actor Chris Hemsworth often cited as an archetypical Chad. Incels suggest that dating apps provide women with sexual/romantic access to a near limitless supply of Chads. They argue Chads use dating apps to pursue sexual relationships with many women simultaneously, leaving most men unpartnered.

Incels ubiquitously rate men’s attractiveness. They identify Chads as the most attractive type of men, followed by normies (or “cucks,” “betamales,” or “numales”), and then incels. While Chads are white, incels identify a subset of men of color as highly desirable, using the terms “Tyrone,” “Chang,” “Chaddam,” to refer to attractive Black, Asian, and Arabic men, respectively. Incels rank all these desirable men above both themselves and normies. In contrast to Witt’s (2020) discussion of incel hostility toward Chads, incels here intimately detail the attractiveness of such men, reflecting on their musculature, height, and jaw-definition, with one user describing a Chad by stating, “his bones radiate so much energy and lust towards women. It’s like he’s a God.” To incels, Chads both command and deserve attention from women.

Incels unfavorably compare their own bodies to those of Chads. While Chad is muscular, tall, and filled with energy, incels perceive themselves as suffering from a lengthy list of physical shortcomings, including thin wrists, small stature, weak jaws, large noses, poor musculature, and misaligned eyes. In contrast to previous studies (Van Valkenburgh 2018), incels are not attempting to become Chads, as part of the “black pill” is accepting their perceived subordinate status and realizing they will never ascend the masculine hierarchy. Incels further suggests that the physical disparities between themselves and Chads drive their sexual isolation:

5-6 s [average looking men] can only date with very low-tier women and that’s because these women are mostly too ugly to even get pumped and dumped [i.e., have a one-night stand] by Chads…. So, as a 5, even if you have 10/10 personality, if you have another deficiency [women] will eliminate you without even looking at your personality.

This user argues that average-looking men with great personalities can only have a sexual or romantic relationship with women who are too unattractive to secure attention from a Chad. To incels, if a Chad shows interest in a woman then incels and “normies” have no prospects with her, even if they have strong personalities in combination with their average looks. In these discussions of Chads, incels orient to a masculine hierarchy (Connell 1995, 2000; Pascoe 2011), wherein men’s bodies are sorted according to their heterosexual desirability.

According to incels, dating apps magnify these masculine disparities, creating a sexual and romantic context wherein incels are unable to compete with Chads, who are transparently and vastly superior. Chads use dating apps to maintain sexual relationships with multiple women and, as one user notes, “Chads [can] basically break all the rules of [pick up artists] in terms of approaching women and it doesn’t matter.” That is, incels argue that Chads are so desirable that they can establish sexual/romantic relationships with women even if they ostensibly violate social norms and dating rules. To demonstrate this, users share the result of a “Tinder experiment,” wherein a fake profile combined the picture of a handsome white man with a biography listing three convictions for sexual assault, an attraction toward minors, and derogatory language toward women. In what incels referred to as an “atomic black pill,” the fake profile successfully matched 79% of the time (162 matches for 204 right swipes). While this “experiment” serves to humiliate women, incels view it as proof that new dating technologies overwhelmingly favor Chads.

Incels acknowledge that apps broaden dating pools, but they suggest this has the counterintuitive outcome of exacerbating inceldom. For incels, the current dating context means that average men, and particularly unattractive incels, have very low odds of finding a sexual partner:

Every girl wants Chad. And chads are like 1/10,000 [of the men in the world]. Each of us need to hit on 10,000 girls before we get a match. You can literally post the secret to creating a nuclear bomb, how to be invisible, or how to make a million dollars in a day, but roasties would rather watch Chad’s face [appear on their social media feeds] then any of that.

Here, dating apps magnify the sexual success of dominant men. Incels situate Chads as willing to have sexual relationships with almost all women, which means other men are simply outcompeted and relegated to involuntary celibacy.

Once again, incels’ discourse connects gender practices, new technology, and the inceldom. In these comments, women compulsively pursue Chads, while Chads rightfully dominate this “winner-take-all” sexual marketplace. Once again, incels’ anger and claims of victimhood are also tied to their sense of entitlement to women’s bodies. While incels direct vitriol at women, Chads receive admiration. It is not Chads’ dominance of women or other men that is the issue, but incels’ perception that, as men, they have been unfairly denied sexual access to women. Incels want to participate in this system, not overturn it.

### Betamales and Instagram

Via social media, incels argue that “betamales” fawn over women, providing them with continuous praise that disrupts a woman’s accurate assessment of her own desirability. Incels suggest that women exploit betamales, while betamale attention leads women to overestimate their physical appeal and believe they “deserve” a Chad as a long-term partner.

Cucks, betamales, and numales are men that incels perceive as subordinate to Chads. While these men can have sexual and romantic relationships, they are always at risk of losing their partner to a more desirable man (e.g., a Chad), and there is a constant threat that their partner is having an affair. As such, incels posit that betamales and numales are often quite literally cuckolds (“cucks”) to Chads. This dynamic is summarized in the oft-repeated slogan “alpha fux, beta bux,” (see also Van Valkenburgh 2018) that describes how betamales provide financial support to women while they are having sexual affairs with more desirable men.

According to incels, betamales are a driving force behind the extensive attention women receive on social media, with hundreds, thousands, or millions of betamales fawning over individual social media accounts. Incels refer to this behavior as “orbiting,” as betamales maintain close proximity to women in the hope that their constant expression of interest and/or financial gifts will increase their chances of establishing a sexual/romantic relationship. In this analogy, the woman is the center of attention around which the betamale “orbits” as he tries to demonstrate his value as a partner and escalate the relationship.

For incels, this orbiting is also pointless. As one user observes, “orbit, orbit and orbit until Chad comes along. Then what are you going to do? Ninety percent of men that orbit fail to get any sex from the female they were orbiting.” Incels argue betamales can never compete with Chads, so orbiting is ultimately a failing strategy. Indeed, incels refer to orbiting as a “trap” that both incels and non-incels can fall into, falsely believing that a woman will reciprocate interest if they provide enough attention. As orbiters are perceived to have been manipulated by women, it is behavior that is starkly critiqued:

She just wants you to give her attention and be an emotional tampon. You have been friendzoned, you aren’t even considered a sexual being in her mind. Why you continue to orbit this whore after she’s made it obvious that she has no interest in you is beyond me.

Here, orbiting is not only a failing strategy, but it is argued that orbiting men are being manipulated by women. This discourse is also highly transactional, as incels label women as exploitative and manipulative when they fail to repay orbiting with sex. Once again, incels explicitly situate themselves, and other men, as entitled to both sex and women’s bodies.

For incels, a crucial part of social media orbiting is that it distorts women’s self-perception of their “sexual marketplace value.” All women, regardless of their attractiveness (which, for incels, is known and quantifiable), receive constant adoration and believe that they are exceptionally desirable. As one user complains, “thirsty [men desperate for sexual contact] pathetic betas” are “responsible for the current state of affairs. At this rate, the pussy price is exponentially growing to infinity.” For incels, sexual relationships are explicitly transactional, with “thirsty betas” artificially increasing the resources women command to engage in sex, which distorts the sexual market. Specifically, betamales follow women on social media and provide them with a steady stream of comments stating how attractive they are. As one user summarizes:

Easy answer [why there are more incels], smartphones. Instant access to every Chad in a 500-mile radius and being showered with admiration and attention 24/7. Every woman is a celebrity with Instagram…Basically, a 2/10 foid gets much more sexual interest in her than a 10/10 did from the 1990s.

Bypassing the amount of critique women receive in these domains (e.g., Jane 2018), this user argues that social media platforms provide women with a steady stream of praise and sexual attention. Indeed, the user estimates that, in the Instagram era, unattractive women receive the praise and adoration that was previously reserved for only the most attractive women.

Incels claim that social media creates an environment wherein women perceive themselves to be highly desirable and they consequently pursue the most attractive men, believing such men to be their rightful “looksmatch.” Incels suggest that women will select casual sex or being single over dating a beta or an incel:

There are actually high beauty standards for men [from women], and women choose to just go on Tinder, or whatever, to have one-night stands [with Chads]. Or stay single. Because they don’t want an average or below average looking guy.

From incels’ perspective, betamale orbiting on social media creates severe distortions in the sexual marketplace, such that women will remain single rather than “under sell” themselves by “settling” with an average or sub-average male partner.

As with dating apps, incels situate social media as revealing and compounding gender practices that produce incels. Chads rightfully dominate, while “pathetic thirsty orbiters” harm all men’s interests by providing women with attention that is not bound to a sexual quid pro quo. For incels, it is not a problem that women are objects, the problem is that they have become overpriced. Once again, this formulation buttresses incels’ essentialist ideas while situating themselves as hapless victims of changing social relations.

## Discussion and Conclusion

As demonstrated in this article, incels argue that inceldom is exacerbated by emerging technologies. First, incels suggest dating apps amplify hypergamy, providing women with access to large numbers of potential mates and facilitating their ability to “upgrade” their partner. Second, incels argue that dominant men (e.g., Chads) can simultaneously pursue numerous women via dating apps, monopolizing sexual and romantic access to women. Third, incels state that men they see as subordinate and submissive (e.g., betamales, cucks) distort women’s perception of their marketplace value through social media orbiting. Here, the “problem” of the inceldom is that new technologies result in an unfair distribution of sexual access to women, producing a hyperstratification of the sexual marketplace. This article provides the first analysis of a popular incel forum, contributes new analytic insights on the experiences of incels, and contributes to the growing literature addressing online communities (Ging 2019; Jane 2018; Nagle 2017; Van Valkenburgh 2018) by demonstrating how incels’ engagement with emerging technology buttresses ideas of hegemonic masculinity, despite their perceived subordinate position in a masculine hierarchy.

Incels are not only making claims about emerging technologies, but they are also making claims about how women act and how they should be treated. Regardless of how women used emerging technologies, incels situated them as repulsive, subhuman, and/or Machiavellian. Indeed, incels situate these technologies as revealing women’s base nature as they enable sexual practices that are unrestrained by male interests, which threatens the social order by creating an ever-increasing number of incels. Incels essentializing discourse on masculinity is employed to both protect and justify misogyny in virtual spaces and beyond. Here, incels are participants in a larger environment of online misogyny, which endorses gender-based hatred (Jane 2018, 2014), while encouraging objectification (Chang 2020), harassment (Moloney and Love 2018), humiliation (Massanari 2017), and manipulation (Van Valkenburgh 2018) of women. Incels might blame emerging technologies for the inceldom, but their anger is firmly levied against women. Despite “taking the black pill” and accepting that “it’s over,” this anger indicates that incels cannot reconcile the dependence of their masculine identity on heterosexual sex with their view that women are inferior.

Incels’ arguments that dating apps and social media exacerbate inceldom combines technological determinism with an essentialist view of gender relations. That is, these emerging technologies are believed to both reveal and compound gender practices, relentlessly increasing the number of men that are involuntarily celibate. As such, incels’ discussions of technology facilitate a relational analysis of their orientation toward gender (Connell 1995). From the incel perspective, women are hypergamous, calculating, and uncaring, as they leverage emerging technology in the pursuit of the ideal masculine partner while maximizing the resources they yield from orbiters. Women are also simultaneously sexual commodities, which can be quantitatively evaluated, subject to market distortions, or be unfairly distributed. Here, incels doubly deny women their humanity, as women are manipulative when acting and overpriced when objectified. While incels accept that they will never attain the status of an alpha, they buttress their own perceived subordination by accepting the black pill, abandoning their sexual/romantic prospects, and avoiding the emasculation characterizing betamales. While Connell (1995) predicts changes to hegemony over time, incels hold fast to a caricatured and essentialist view of gender, with novel technologies amplifying the existing gender dynamics, providing even starker contrasts between the winners and losers in masculine hierarchies. However, resonating with Connell, incels do perceive changes in gender relations, viewing women as developing increasing control over their mate selection, to the detriment of men and their ability to control women.

While our analysis of incels draws upon Connell’s (1995) masculinities theory, prior analyses of online communities have employed the concept of hybrid masculinities (Ging 2019), which details how dominant men selectively adopt practices, traits, or characteristics from marginalized groups (e.g., Bridges and Pascoe 2014; Demetriou 2001; Messner 2007). We did not identify similar hybrid practices in our analysis of incels, who instead asserted a caricatured view of alphas, betas, and women. However, incels’ avoidance of hybrid practices, in combination with their essentialized descriptions of dominant men, provides an analytic foil for hybrid masculinity. Specifically, as incels label themselves as subordinate men, men enacting hybrid masculinity might also be working to distinguish themselves from such sexually unsuccessful men. Incels enactment of a coarse-grained version of hegemonic masculinity might be characteristic of men who are experiencing diminishing patriarchal returns (Connell 1995), while the men who continue to command social resources, such as the men incels admire, have instead transitioned to hybrid masculine practices. Future research could investigate whether hybrid masculine performances are strategically positioned in relation to the practices of men who are sexually unsuccessful and hostile toward women (e.g., incels). Indeed, just as men use “fag” to police and evaluate masculinity (Pascoe 2011), in online communities they employ “have sex” or “have sex, incel” to dismiss the points made by other men or to suggest that their opinions are similar to those of incels. Consistent with hybrid masculine practices, this insult also serves to differentiate the men using it from incels and their openly misogynistic stances.

Incels, in terms of both their current and emerging practices, demonstrate the relevance of continued social scientific research on online communities (see also Baele, Brace, and Coan 2019; Nagle 2017; O’Neill 2018; Jane 2018; Ging 2019; Schmitz and Kazyak 2016; Van Valkenburgh 2018), even if such communities have antisocial or offensive practices. Indeed, some of the claims incels connect to emerging technology are documented in social science research. Many men are unsuccessful on dating apps (Rudder 2009), there are social biases in favor of attractive people (Dion, Berscheid, and Walster 1972), and more men are reporting diminished or absent sexual activity (Ingraham 2019). While incels’ observations are accurate, their explanations for these observations are grounded in reductive and dehumanizing descriptions of both men and women. Many men also face similar challenges but do not endorse misogyny. Here, incels assert a distorted sociological imagination (Mills 1959), in that they have connected their personal troubles to broader social issues, but in a manner that reinvigorates hegemonic ideals and practices (Connell 1995), such as the tight coupling of heterosexual sex and masculine worth, a rigid hierarchy amongst men, and discomfort with women’s agency.

The window for any substantial social analysis of incels is also considerably narrow. On one hand, incels face the prospect of medicalization (Conrad 2005), a process by which forms of deviance become the domain of medical professionals. In this context, membership in the incel community might be indicative of mental illness (Duong 2020). On the other hand, incels face the prospect of criminalization, as their communities are increasingly seen as violating laws and engaging in domestic terrorism (e.g., CBC 2019). The criminalization of incel communities is particularly salient after incel-associated mass murders, such as those in California, Ontario, and Georgia, the latter of which led to the site we analyze being shut down and forced to migrate to a different web address. As such, continued research on incels is important given that the community’s web presence might be formally or informally prohibited or incels might instead find ways to move their discussions to more obscure locations that are outside of the purview of institutions, authorities, and social scientists.

There are limitations to the study. First, the study analyzes online data, so we cannot verify information about users. While analyzing incels offline would be difficult, the study remains dependent on the accuracy of users’ self-reports. Second, while social desirability bias might have less of an influence on incels’ online interactions with each other, individual’s online behaviors might or might not reflect their in-person behavior, and, accordingly, incels might not endorse their online views during in-person interactions. Lastly, a subgroup of incels we observed did not display hostile attitudes toward women (or any other group), criticized Elliot Rodger, and critiqued such attitudes among other incels. These incels situate inceldom as a social problem that cannot be addressed if incels continue antisocial actions. The perspectives of these men warrant an independent analysis.
